# Threshold Behavior Hidden in the Growth Response of Peat Moss *Sphagnum riparium* to Temperature

**DOI:** 10.3390/plants13223241

**Published:** 2024-11-19

**Authors:** Victor L. Mironov

**Affiliations:** Institute of Biology of the Karelian Research Centre of the Russian Academy of Sciences, Pushkinskaya St. 11, 185910 Petrozavodsk, Russia; vict.mironoff@yandex.ru

**Keywords:** mires, *Sphagnum* mosses, growth monitoring, temperature threshold, thermal optimality, photosynthesis, respiration, carbon retranslocation

## Abstract

The balance between photosynthetic carbon accumulation and respiratory loss in plants varies depending on temperature. This leads to a situation where the increased need for carbon is not met when a certain temperature threshold is reached. Over the last two decades, temperature thresholds in carbon metabolism in autotrophic systems have been widely studied. However, it remains unclear how these thresholds manifest themselves in the natural growth of individual plant species. To address this issue, we used data from an extensive monitoring of the growth of peat moss *Sphagnum riparium* over 9 years in mires in Karelia (Russia). We measured the growth of shoots in sample plots and obtained 1609 estimates of growth rates during the monitoring period. Investigating the relationship between growth rate and temperature, we identified two distinct intervals in response to temperature. These two intervals are separated by the temperature threshold of 13.2 °C. The first interval, which covers 42% of the growing season, exhibits a strong exponential dependence of growth rate on temperature, with a coefficient Q_10_ = 4.01. This indicates that growth is most sensitive to changes in temperature within this range. In contrast, the second interval (58% of the growing season) shows a weaker dependence, with a Q_10_ coefficient of 1.21, suggesting that growth is less responsive to changes within this temperature range. The temperature threshold was found to be negatively related to May (r = −0.76; *p* = 0.018) and September (r = −0.78; *p* = 0.012) temperatures of the previous growing season, and together they best explain (r = −0.91; *p* = 0.0007) the temperature threshold. Overall, our findings suggest that the temperature threshold does exist in the growth of *S. riparium* and can be identified in different years. The negative correlation between temperature threshold and May and September temperatures from the previous year indicates that intervals in the growing season with temperatures near the temperature threshold have an impact on subsequent carbon balance and are particularly significant for the further growth and development of *Sphagnum* mosses.

## 1. Introduction

According to the van’t Hoff rule, as the temperature increases, plants are able to use exponentially increasing amounts of carbon in their metabolism. However, one of the main challenges for this is that the balance between photosynthetic production and respiratory losses changes with increasing temperatures [1,2]. At low temperatures, plants produce more carbon through photosynthesis than they emit through respiration. Therefore, the plant’s metabolic processes are not limited by a shortage of carbon. However, as temperatures rise, respiratory losses begin to equal photosynthetic production, meaning that, despite continued increases in photosynthetic productivity, there is insufficient carbon available for the plant’s metabolic needs at a certain temperature. This phenomenon, known as the temperature threshold [3,4], occurs when a plant’s demand for carbon exceeds its supply.

In recent years, researchers have identified temperature thresholds in individual plant species [5], plant communities [6], ecosystems [7,8,9] and the biosphere as a whole [2]. Among these autotrophic systems, temperature thresholds manifest themselves not only directly in carbon metabolism, but also secondarily in the respiration process [3,10]. In northern latitudes, the temperature thresholds typically range from 10 to 20 °C, which is close to the average temperature during the growing season [8]. This proximity is thought to be due to the plants’ adaptation of photosynthesis and respiration to the typical temperature conditions in their environment [7,8]. At the same time, climate warming stimulates a gradual increase in temperature thresholds in the long term [8]. Although temperature thresholds have been studied primarily in the context of the thermal optimality of carbon metabolism, our knowledge of how they influence plant growth processes is still limited.

Growth is one of the most visible indicators of photosynthetic activity in plants [11]. The close relationship between these parameters is based on the fact that plant cells and tissues that are formed as a result of division of meristematic cells act as attractors for carbon absorbed during photosynthesis [12]. Since there is threshold behavior in the temperature dependence of carbon metabolism in autotrophic systems, it is reasonable to assume that there is also threshold behavior in the temperature dependence of various plant growth characteristics. The simplest of these characteristics is shoot growth rate, which can be accurately measured by observing plant growth in nature over a long period of time. Plants that grow continuously throughout the growing season and are exposed to a wide range of temperatures are best suited for such observations.

In northern latitudes, one example of such plants is peat moss of the genus *Sphagnum*. These mosses are key dominants and peat-forming agents in raised and transitional bogs [13]. They have continuous apical growth which begins immediately after spring thaw and stops when autumn freezing sets in. The lower parts of their shoots continuously decompose, resulting in dead parts that often exceed the length of living parts by several times. The relationship between *Sphagnum* growth and temperature has been studied in numerous papers [14,15,16,17,18,19,20,21,22,23,24,25]. However, there has still been no report of temperature thresholds for their growth.

Since 2015, we have been conducting extensive monitoring of the growth of the peat moss *Sphagnum riparium* Angstr. (Sphagnaceae, Bryophyta) in the mires of Karelia (Russia) [23,25,26,27,28]. This monitoring is carried out with an average interval of two days between observations, and currently covers nine complete growth seasons. During this time, we have measured an increment of 269,945 shoots and obtained 1617 estimates of growth rates. We recently discovered a strong temperature dependence on the growth rate of *S. riparium* [27], which varies depending on geomagnetic conditions [25]. Our current data set also allows us to test the hypothesis of a threshold behavior in the response of *S. riparum* growth rate to temperature.

## 2. Materials and Methods

### 2.1. Study Location

The study site included two mires located in the middle taiga of southern Karelia (Russia), near the city of Petrozavodsk. Their detailed description is presented in our previous papers [23,26,27,28]. The study was conducted on a mire site of 0.12 hectares (N 61°51′14″; E 34°10′51″; 50 m a.s.l.) from 2015 to 2018. *Sphagnum* mosses cover almost the entire mire surface. Among them, *S. riparium* occupies more than 90% of the site, and *S. squarrosum, S. divinum, S. centrale, S. fallax* and *S. angustifolium* occupy up to 7% of the site. Among vascular plants, *Salix phylicifolia, Equisetum fluviatile, Calamagrostis canescens, Comarum palustre, Carex rostrata, Calla palustris* and *Typha latifolia* were the most common, covering from 5 to 30% of the site. In 2018, research on this mire site was stopped due to its reclamation. In 2019–2023, the study was continued in the drainage ditches of the mire near Dennaya Lamba (N 61°44′39″; E 34°16′05″; 160 m a.s.l.), which were similar in humidity to the previous mire. The width of these ditches was 1.5–2.0 m, the length was 400–600 m and the depth was 0.5–1.5 m. The plant cover in the ditches was uniform and consisted of a continuous cover of *S. riparium* with a minor presence of *S. fallax* and *S. angustifolium*, as well as sedges *Carex rostrata* and *C. magellanica*.

### 2.2. Study Object

The object of our study is *S. riparium*, a species of peat moss that has a circumpolar distribution in Europe, Asia and North America. It is typically found in flooded mires, reclamation ditches of bogs and secondary flooded paludificated areas. In these habitats, *S. riparum* forms extensive, smooth carpets that can grow several tens of centimeters each year [24,29]. During most of the growing season, these carpets are kept moist, so complete drying and growth cessation are not typical for *S. riparium*. Therefore, this species maintains the ability to respond to environmental stimuli throughout the growing season.

### 2.3. Experimental Design

In this study, we used data obtained over nine full growth periods of *S. riparium*. Every year, between 3 and 13 sample plots with dimensions of 3 × 3 and 5 × 1 m^2^ in the first and second mires, respectively, were used in the study. The plots were established on flat and intact areas of *S. riparium* with up to 15% vascular plant cover.

**Figure 1:**
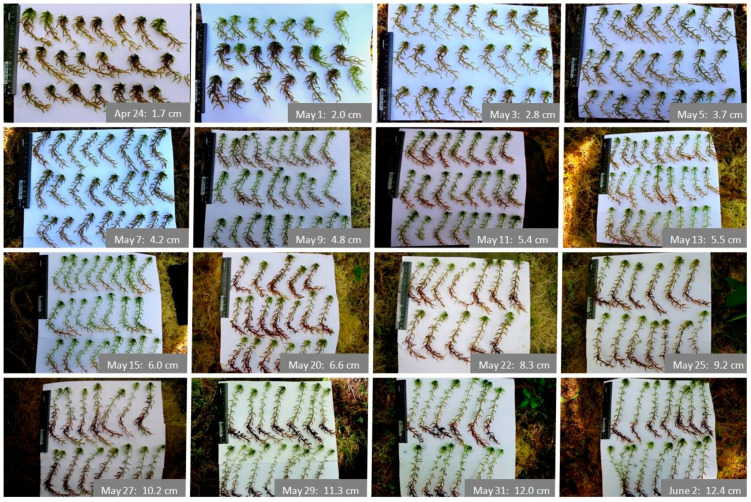
An example of the growth dynamics of *Sphagnum riparium* on one of the sample plots from 24 April to 2 June 2016. The graph shows the growth in relation to the distance from the nival geotropic curvature to the tip of the shoot. The captions inside the figure provide information on the average growth of plants in the sample plot.

We measured the linear growth of *S. riparium* using the geotropic curvature method, which has been described in detail in previous papers [23,27,29]. Nival and artificial curvatures of the stem induced by snow load and artificial indentation of the *Sphagnum* cover were used as markers for growth. Nival curvatures were used at the beginning of the season, when they naturally appear on the shoots after snow melts (Figure 1). After the shoots had grown, we used artificial curvatures to reduce measurement error. We achieved this by applying a brief pressure using a plywood sheet on the *Sphagnum* cover. Within 1–3 days, these artificial curvatures formed on the shoots, which we used as new growth markers. The induction of artificial curvatures was repeated several times throughout the season, typically 3–6 times. During monitoring, we took sequential samples of shoots from small fragments of *S. riparium* cover (approximately 100 cm²) within each sample plot and measured their linear growth [23]. Each subsequent sample was taken from an undisturbed area of the cover, several centimeters away from the previous sample location. Based on the difference in average increments before and after each sampling interval, growth rates were calculated for each sample plot (Figure 1). Subsequently, by averaging the growth rate patterns across all sample plots, we obtained growth patterns for the entire mire area, which were then used for further analysis.

### 2.4. Temperature Data Sources

In this study, we used soil surface temperature data collected at the Petrozavodsk weather station (WMO ID 22820). The weather station is located near the studied mires, approximately 4.5 km and 8.2 km away from the first and second mire, respectively. We have previously shown that soil surface temperature at this station correlates better with the growth rate of *S. riparium* than air temperature [25]. Additionally, the correlation between soil surface temperature and the growth of *S. riparium* shows a clearer diurnal bimodal pattern compared to air temperature [25]. As bimodal patterns are common in many plant processes in natural environments, this finding supports our decision to use soil surface temperature as a parameter. The data source for soil surface temperatures is the AISORI network remote access technology, which can be accessed via the following link http://aisori-m.meteo.ru/waisori/ (accessed on 14 April 2024). Here are the temperature values at 3 h intervals (0:00, 3:00, 6:00, 9:00, 12:00, 15:00, 18:00 and 21:00) that we used for our analysis. Based on them, we calculated the average daily temperatures that were used in our analysis.

### 2.5. Statistical Analysis

Before data analysis, *S. riparium* growth rate patterns were processed using a simple 3-day moving average to reduce the impact of random non-dominant variations caused by extrapolating a single value across the entire interval between observations [23].

We then analyzed the overall temperature dependence of *S. riparium* growth rate. To do this, we tested the linear and exponential models for the scatterplot. Initially, we excluded several negative growth rate estimates from the analysis. After that, the scatterplot was processed with a moving average of 100 data points to reveal hidden patterns. This moving average dimension was chosen empirically, as it was the smallest size that clearly demonstrated the pattern of growth response to temperature. The moving average processing consisted of two stages. First, all growth rate values were sorted from minimum to maximum. Then, the average growth rates and corresponding temperatures were calculated in a moving window. This allowed us to trace the hidden dynamics of the *S. riparum* growth response to temperature within the scatterplot and identify the temperature threshold. The detection of the temperature threshold was conducted in three stages. Firstly, we visually identified the temperature at which the exponential growth rate began to weaken. Secondly, we used data points below and above that transition temperature to create exponential trend lines. Finally, we adjusted the amount of data points above and below the transition temperature to achieve the point where the two trend lines intersect. This point represents the temperature threshold.

One of the objectives of our study was to assess how the temperature threshold for *S. riparium* growth changed in different years of the study. The analysis was similar to the previous one, but we used a moving average of 20 data points. This was the smallest time frame for which we could identify the temperature threshold in all years. Due to the fact that the patterns for each individual year were more complex than the overall pattern, the temperature threshold was determined by visually identifying the point where the relationship between growth rate and temperature started to gradually decrease.

The influence of temperature conditions from the previous vegetation period on the current temperature threshold was estimated in the following way. First, the average temperature for each month (April to October) from the previous growing season was calculated. Then, the correlation of these temperatures with the temperature threshold of the current vegetation season was determined. We used the Pearson correlation coefficient for this analysis, but for greater confidence in our results, we also calculated the non-parametric Spearman and Kendall coefficients. These gave similar results, so they are not presented here. We set a significance level of *p* = 0.05 for our analysis.

## 3. Results

### 3.1. Brief Description of the Data Collected from Growth Monitoring

A brief description of the growth monitoring of *S. riparium* is presented in Table 1. Over the course of 9 years, a total of 725 days were spent collecting field material. During this time, the growth of 226,945 shoots was measured, resulting in 11,155 estimates of growth rate in sample plots and 1617 in the mire. Thus, each growth rate estimate for the mire was based on measuring the growth of approximately 140 shoots.

The average growth rate by year was as follows: 2015—0.23 ± 0.10 cm day^−1^ (mean ± s.d.), 2016—0.21 ± 0.13 cm day^−1^, 2017—0.22 ± 0.12 cm day^−1^, 2018—0.21 ± 0.11 cm day^−1^, 2019—0.28 ± 0.16 cm day^−1^, 2020—0.16 ± 0.09 cm day^−1^, 2021—0.16 ± 0.10 cm day^−1^, 2022—0.19 ± 0.10 cm day^−1^ and 2023—0.20 ± 0.08 cm day^−1^. The average growth rate for the entire study period was 0.20 ± 0.12 cm day^−1^.

**Figure 2:**
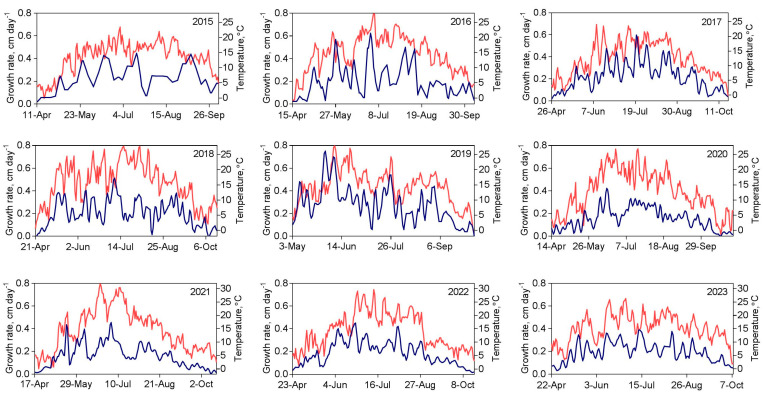
Growth rate and temperature patterns of *Sphagnum riparium* for the study period. The blue line represents the growth rate and the red line represents temperature.

The seasonal patterns of *S. riparium* growth rate and temperature are shown in Figure 2. As seen, the growth rate of the moss varied significantly throughout the season in different years but generally followed the seasonal trend of temperature, consistent with our previous findings [23].

### 3.2. Temperature Dependence of the Growth Rate Based on Linear and Exponential Models

**Figure 3:**
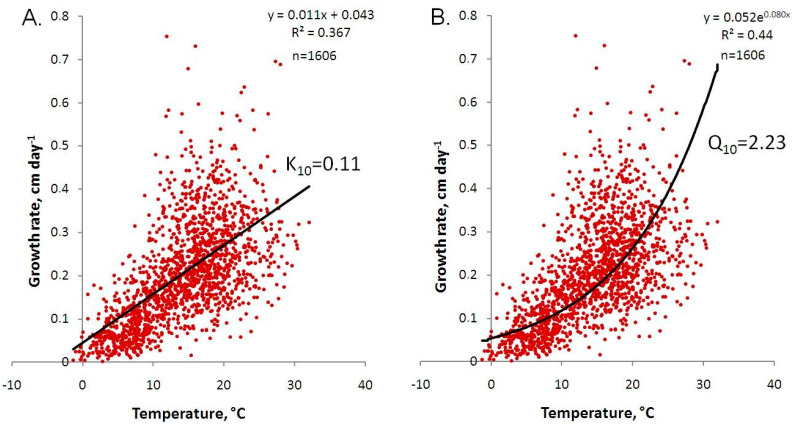
Temperature dependence of *Sphagnum riparium* growth rate based on linear (**A**) and exponential (**B**) models.

The initial analysis showed that a 10 °C increase in temperature leads to a 0.11 cm day^−1^ increase in the growth rate of *S. riparium*, according to the linear model (n = 1606, R^2^ = 0.37), and a 2.23-fold increase according to the exponential model (n = 1606, R^2^ = 0.45). These results are presented in Figure 3. The estimates obtained from these two models are very close to those previously obtained based on four years of research [23].

### 3.3. Temperature Dependence of the Growth Rate, Based on a Moving Average, Reveals Temperature Threshold

**Figure 4:**
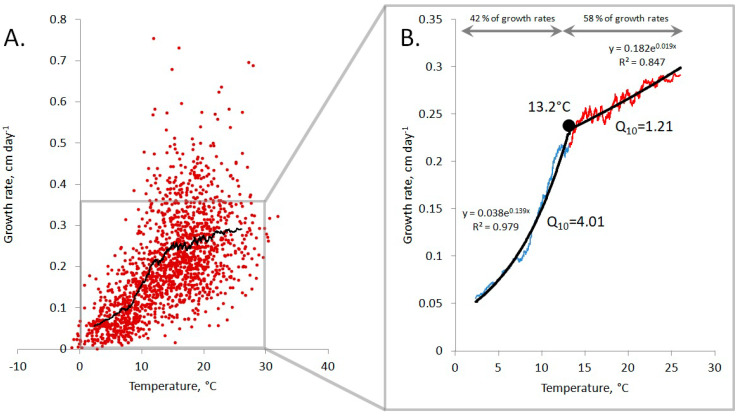
Temperature dependence of the growth rate of *Sphagnum riparium* based on moving average. (**A**) represents a moving average of 100 data points, and (**B**) shows the intervals with different levels of temperature sensitivity revealed by the analysis. The blue line indicates the first interval, which has the highest level of temperature sensitivity, while the red line represents the second interval, with reduced sensitivity. The point at 13.2 °C corresponds to the temperature threshold.

Processing with a moving average of 100 values has shown that the growth rate of *S. riparium* has a more complex and heterogeneous relationship with temperature than the linear and exponential models suggest. This relationship is illustrated in Figure 4, which shows two distinct intervals with different responses to temperature changes. The transition between these intervals occurs at 13.2 °C, corresponding to the temperature threshold. This threshold is close to the average temperature during the growing season (14.2 °C).

The first interval includes growth rate values under thermal conditions below the temperature threshold and accounts for 41.8% of all growth rates. The moving average indicates that the temperature response in this range closely follows the exponential dependence. This dependence is significantly stronger (Q_10_ = 4.01) compared to the one obtained by approximating the entire data set (Q_10_ = 2.23), suggesting that the model based on the entire data set underestimates the impact of temperature on the growth rate of *S. riparium* within this interval.

The second interval includes growth rate values under thermal conditions above the temperature threshold and covers 58.2% of all growth rate values. A characteristic feature of this interval is a weakened positive temperature response. The change in this response across the entire interval can be described by linear and exponential functions approximately equally well. An increase in temperature by every 10 °C leads to an increase in the growth rate of *S. riparium* by 0.05 cm day^−1^, according to the linear model, and by a factor of 1.21, according to the exponential model. This suggests that the full data set model overestimates the effect of temperature on the growth rate of *S. riparium* within this interval.

### 3.4. Temperature Threshold in Different Years

**Figure 5:**
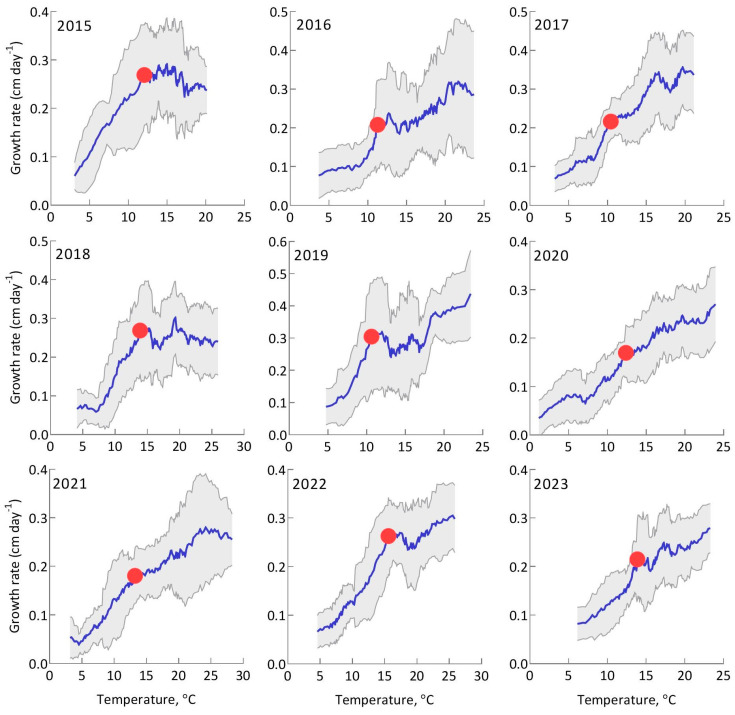
Temperature dependence of the growth rate of *Sphagnum riparium* based on moving average over different years. The blue line represents the moving average of 20 data points, and the gray shading indicates the standard deviation of these values. The red dot indicates the temperature threshold.

The dependence of the growth rate of *S. riparium* on temperature and the temperature thresholds in different years are presented in Figure 5. Due to the fact that each growing season includes a limited number of growth rate values (between 162 and 205), non-temperature variations are not always balanced during the season, resulting in the first and second intervals not being as pronounced in different years as they are in the general dependence. However, by using a moving average of 20 values, it was possible to identify temperature thresholds in each year. Relatively weak differences in the temperature dependence of the growth rate were observed between the first and second intervals in 2020 and 2021, while obvious differences were present in 2016, 2017, 2019, 2022 and 2023. The temperature dependence in the second interval showed a negative slope in 2015 and 2018.

### 3.5. Dependence of the Temperature Threshold on the Temperatures of the Previous Growing Season

**Figure 6:**
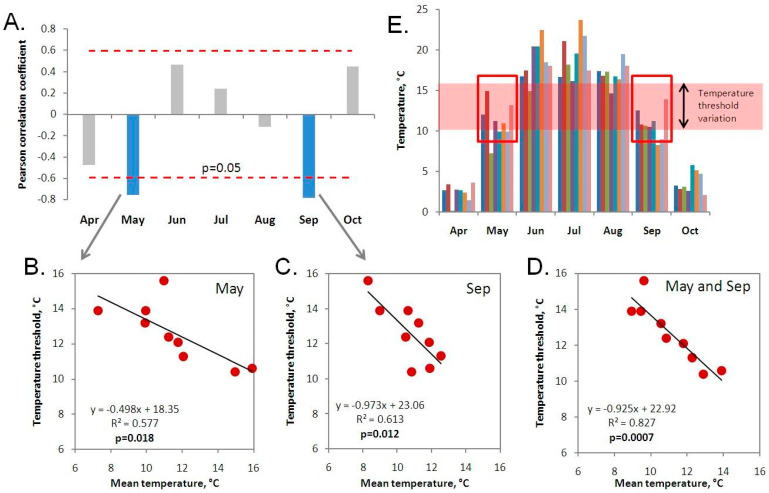
Dependence of the temperature threshold on the temperatures of the previous growing period. (**A**) Pearson correlation between the average temperature of different months and the temperature threshold in the following year. Dependence of the temperature threshold on the average temperatures of May (**B**), September (**C**), and the average temperature between May and September (**D**). (**E**) Average temperatures of individual months in each vegetation period (temperatures are presented in order from 2015 to 2023). The red box shows that, in May and September, the variation in average temperature best overlaps the variation in the temperature threshold.

We analyzed the influence of the average temperature during the previous growing season and the average monthly temperatures on the subsequent temperature thresholds. The results are presented in Figure 6. The analysis showed that the average growing season temperature does not significantly affect the temperature threshold (r = −0.38; *p* = 0.31). We also found that the threshold does not depend on average temperatures in April (r = −0.47; *p* = 0.19), June (r = 0.47; *p* = 0.21), July (r = 0.24; *p* = 0.53), August (r = −0.12; *p* = 0.76) or October (r = 0.45; *p* = 0.22). However, the analysis revealed a negative correlation between the temperature threshold and mean temperature in May (r = −0.76; *p* = 0.018) and September (r = −0.78; *p* = 0.012), with the mean temperature between these months being the strongest predictor for the threshold in the next growing season (r = −0.91; *p* = 0.0007).

## 4. Discussion

Temperature is a crucial factor for the growth of plants [11], including *Sphagnum* mosses [15,16,17,18,19,20,21,22,23,24,25]. The free carbon pool within the plant is a limiting factor for an adequate growth response to temperature [12,30]. If this pool exceeds the amount that the plant can metabolize at a certain temperature, then the temperature growth response will be most effective. Conversely, if the pool is lower than the amount required for metabolism at that temperature, it will weaken the temperature growth response.

Numerous studies have shown that optimal carbon exchange in natural autotrophic systems occurs at lower temperatures than photosynthesis would predict [2,5,6,7,8,9]. This effect is thought to be due to respiratory losses, which increase continuously with increasing temperature [2]. These losses have little impact on the free carbon pool at lower temperatures, but they greatly reduce it at higher temperatures. As a result, plants experiencing intense photosynthesis under high temperatures may experience carbon deficiency, leading to inadequate carbon exchange under these conditions.

It was unclear whether the limitation of carbon metabolism is reflected in the natural growth response of plants to temperature. Growth studies conducted under controlled laboratory conditions did not seem to support this viewpoint, as they showed that a decrease in growth rate was observed at relatively high temperatures, which is approximately the same temperature at which photosynthesis decreases [31,32]. However, our nine-year field monitoring of *S. riparium* growth, on the contrary, supported this viewpoint. We found that the species’ growth response to temperature has two distinct intervals separated by the temperature threshold. In the first interval, there is a strong exponential response to temperature (Q_10_ = 4.01), while in the second interval, this response suddenly weakens (Q_10_ = 1.21). The temperature threshold (13.2 °C) is close to the average temperature of the growing season (14.2 °C), which has previously been noted in optimal carbon exchange [8]. Our data are consistent with the hypothesis that *S. riparium* growth receives sufficient carbon support for an adequate temperature response in the first interval, but insufficient carbon support in the second interval.

One of the distinctive features of our study was that, despite the significant weakening of the growth rate response of *S. riparium* at temperatures above the temperature threshold, it still maintained a positive trend. This finding seems anomalous, as it contradicts the expectation that increased respiratory losses, combined with natural temperature limitations on photosynthesis, should lead to progressive depletion of the carbon pool [2]. As a result, this should lead to a decrease in *S. riparium* growth rates with increasing temperatures. Experimental data from autotrophic systems, both at the species and community levels, support this expectation, as a progressive decrease in carbon exchange is typically observed above the optimum temperature [5,6,8]. However, in our study, we did not observe a similar decrease in the growth rate of *S. riparum*. The most plausible explanation for this discrepancy is that previously accumulated carbon may have been involved in shoot growth. Its main source of carbon in *Sphagnum* mosses is the dying lower parts of the shoots, which are sometimes several times longer than the living parts. These dying parts serve as a natural storage for carbon and other biogenic elements in the *Sphagnum* [33,34]. Therefore, acropetal retranslocation can maintain a positive carbon balance for growing shoots over long periods, despite increased respiratory losses. The use of stored carbon by plants with high respiratory losses has been demonstrated both theoretically and practically [35]. This is also indirectly supported by the fact that the upward movement of metabolites in plants increases at higher temperatures, and these processes have a high temperature coefficient Q_10_ [36].

Our study leaves unclear the extent of the effects of an unfavorable carbon balance on the long-term growth of *Sphagnum* plants at temperatures above the temperature threshold. These effects may be limited solely to processes associated with growth, but we cannot exclude the possibility that they may have broader effects, affecting the carbon content of *Sphagnum* cells and tissues. A recent study by Shtang et al. [37], conducted in the ombrotrophic mire system Ilasskoe near Arkhangelsk (Russia), found that the carbon content of four species of *Sphagnum* was significantly lower in June (36.40—38.61%) compared to September (45.30–45.69%). Additionally, the cellulose content of the shoots was also lower in June (21.26–30.09%) compared to September (60.54–68.05%). Given that *Sphagnum* experiences higher temperatures in June, these observations [37] could be attributed to carbon deficiency resulting from prolonged growth under relatively warm conditions.

The temperature threshold for the growth rate of *S. riparium*, found here, along with previous data on the optimal temperature for carbon metabolism in ecosystems [6,7,8], raises a reasonable question: why are temperature thresholds undetectable in the laboratory, while they are clearly visible in nature? One possible explanation for this discrepancy is that the balance between respiratory losses and photosynthetic carbon accumulation is different in these two environments. In the laboratory, respiratory losses mainly occur through dark respiration, which releases about 40–45% of photosynthetically acquired carbon. The ratio of dark respiration to photosynthesis remains relatively constant over a wide range of temperatures [38], except at higher temperatures, where it increases significantly [39]. However, in natural conditions, respiratory losses also occur through photorespiration. Photorespiratory losses in plants usually account for 20–40% of the carbon absorbed during photosynthesis [40,41,42]. Under stress conditions, such as high light, high temperatures, or lack of moisture, these losses can become significantly greater, leading to more intense respiratory losses. Therefore, higher levels of respiratory losses under natural conditions can lead to a carbon deficit at moderate temperatures, which can cause the occurrence of the temperature threshold. This occurrence may also be partly due to the lower efficiency of plant photosynthesis in natural ecosystems compared to laboratory conditions. The decrease in photosynthetic efficiency may occur due to photoinhibition, which is a common phenomenon in *Sphagnum* under natural conditions, even with moderate sunlight [43,44]. Since this negative effect significantly reduces the production of *Sphagnum*, it should also increase the ratio between respiratory losses and photosynthetic accumulation. Another major limitation of photosynthesis in these mosses is the decrease in water content in the shoots [45]. In natural conditions, the water content in shoots usually decreases during the summer, which can also reduce photosynthetic activity. Therefore, decreased photosynthetic carbon accumulation combined with increased respiratory losses seems to be a likely cause of the occurrence of the temperature threshold in natural conditions.

The data collected here allowed us to examine how temperature conditions during the growing season affect the temperature threshold for *S. riparium* growth rate the following year. Recent studies suggest that there is a gradual shift in temperature thresholds due to climate warming [8,9]. Therefore, it was initially anticipated that temperatures in certain months would have a positive impact on temperature thresholds the following year. However, the analysis revealed a significant negative influence of average temperatures in May (r = −0.76, *p* = 0.018) and September (r = −0.78, *p* = 0.012). The average temperature between these two months had the strongest impact on the temperature threshold (r = −0.91, *p* = 0.0007). According to the linear regression analysis, a 1 °C decrease in the average temperature in May resulted in an increase of 0.50 °C in the temperature threshold for the next growing season, and a similar decrease in September temperature resulted in an increase of 0.97 °C in the temperature threshold. However, in other months and during the entire growing season, temperature did not have a significant effect on the temperature thresholds for the following year. It is possible that the reason why only May and September temperatures affected the temperature thresholds is because these are the only temperatures that varied near the thresholds during the *S. riparium* growing season. The average May temperature ranged from 7.3 to 15.0 °C, while the average September temperature ranged from 8.3 to 13.9 °C. The temperature threshold ranged from 10.4 to 15.6 °C during this time period.

Our findings suggest that the stages of the previous growing season, with temperatures close to the temperature threshold, leave an imprint on the subsequent carbon balance and significantly affect the further growth and development of *S. riparium*. The effect of September temperatures is likely due to their hardening effect [46]. Hardening of plants due to lower temperatures (10–15 °C) has several physiological effects, including the direct prerequisite for a subsequent increase in the threshold temperature. This includes the formation of additional carbohydrate reserves and a shift in photosynthetic productivity toward lower temperatures [46,47,48]. However, the cause of the impact of May temperatures remains unclear, as these temperatures and their response were always separated by a long period of high temperatures. One possible explanation for the phenomenon is that May temperatures may have hardened the shoots with gametangia, where reproductive cells are formed. If the sporogons formed after the fusion of these cells and then the spores retained information about the hardening event, then the shoots that grew from them and were incorporated into the *Sphagnum* mat during the next growing season could potentially be influenced by the May temperature from the previous growing season.

The present study is the first step in our investigation of temperature thresholds in the growth of *Sphagnum* mosses. This study focuses on their general characteristics, analyzing variation over time and examining dependence on previous temperature conditions. Since temperature thresholds are determined by a balance between photosynthetic carbon accumulation and respiratory loss, we expect that various factors, such as daylight duration, moisture availability and the CO_2_/O_2_ ratio, will significantly influence them. While we have not investigated these factors in this study, the first half of the growing season typically features longer daylight hours, higher moisture content and an increased intake of CO_2_ through heterotrophic respiration compared to the second half. In addition, the plants had been hardened by September temperatures at the end of the previous growing season. Therefore, we hypothesize that these factors may shift the optimal photosynthesis level, and hence the temperature threshold, toward lower temperatures during the first half of the growing season. At the same time, the shorter daylight hours and increased moisture deficiency in the second half of the season contribute to a more significant increase in respiratory loss compared to the first half. This creates the prerequisites for a shift in the temperature threshold towards higher temperatures in the second half of the growing season. Further research is required to accurately determine how temperature thresholds respond to different environmental factors.

In conclusion, our study confirmed the hypothesis that there is a threshold behavior in the response of *S. riparium* growth to temperature. We found that the temperature threshold for our full data set was located at 13.2 °C, but it varied between 10.4 and 15.6 °C in different years. These findings complement our understanding of the influence of temperature on plant production processes and its impact on temperature thresholds. Previously, Niu et al. [8] showed that, over time, the temperature thresholds for ecosystems gradually shift toward higher temperatures, which is associated with plants’ adaptation to a warming climate. However, there is currently a lack of data regarding how temperature will impact temperature thresholds in the medium term. The inverse relationship between May and September temperatures and the temperature threshold for *S. riparium* growth in the next growing season provides some of the first evidence in this direction. We attribute this relationship to cold hardening, although it remains to be determined whether it is solely a result of the change in carbon balance, or if it also provides benefits for the growth and survival of *S. riparium*.

## Figures and Tables

**Table 1:** Key characteristics of the growth monitoring of *Sphagnum riparium*.

	2015	2016	2017	2018	2019	2020	2021	2022	2023	2015–2023
Number of sampling events, days	34	68	88	89	77	102	92	90	85	725
Number of sample plots	4	11	13	6	3	10	10	10	8	3–13
Number of shoots measured	9087	30,267	45,278	31,837	10,526	34,195	24,000	24,300	17,455	226,945
Number of growth rates from sample plots	530	1365	1578	1020	544	1706	1608	1608	1196	11155
Number of growth rates from mire area	178	178	178	180	156	205	184	180	170	1617
Mean sample size (±SD), shoots	93.7 ± 29.6	59.7 ± 15.8	57.8 ± 16.7	60.0 ± 23.0	46.0 ± 6.1	39.9 ± 0.6	30.0 ± 0.0	30.0 ± 0.0	30.0 ± 0.0	30–94
Mean interval between sampling events (±SD), days	5.2 ± 1.5	2.8 ± 0.9	2.0 ± 0.0	2.1 ± 0.2	2.1 ± 0.5	2.0 ± 0.1	2.0 ± 0.23	2.0 ± 0.0	2.0 ± 0.0	2.0–5.2

## Data Availability

The author declares that all data supporting the findings of this study are available in the paper.
